# Effects of Adhesion Dynamics and Substrate Compliance on the Shape and Motility of Crawling Cells

**DOI:** 10.1371/journal.pone.0064511

**Published:** 2013-05-31

**Authors:** Falko Ziebert, Igor S. Aranson

**Affiliations:** 1 Physikalisches Institut, Albert-Ludwigs-Universität, Freiburg, Germany; 2 Institut Charles Sadron, Strasbourg, France; 3 Materials Science Division, Argonne National Laboratory, Argonne, Illinois, United States of America; 4 Engineering Sciences and Applied Mathematics, Northwestern University, Evanston, Illinois, United States of America; University of Zurich, Switzerland

## Abstract

Computational modeling of eukaryotic cells moving on substrates is an extraordinarily complex task: many physical processes, such as actin polymerization, action of motors, formation of adhesive contacts concomitant with both substrate deformation and recruitment of actin etc., as well as regulatory pathways are intertwined. Moreover, highly nontrivial cell responses emerge when the substrate becomes deformable and/or heterogeneous. Here we extended a computational model for motile cell fragments, based on an earlier developed phase field approach, to account for explicit dynamics of adhesion site formation, as well as for substrate compliance via an effective elastic spring. Our model displays steady motion vs. stick-slip transitions with concomitant shape oscillations as a function of the actin protrusion rate, the substrate stiffness, and the rates of adhesion. Implementing a step in the substrate’s elastic modulus, as well as periodic patterned surfaces exemplified by alternating stripes of high and low adhesiveness, we were able to reproduce the correct motility modes and shape phenomenology found experimentally. We also predict the following nontrivial behavior: the direction of motion of cells can switch from parallel to perpendicular to the stripes as a function of both the adhesion strength and the width ratio of adhesive to non-adhesive stripes.

## Introduction

Substrate-based cell motility is involved in many vital biological processes like morphogenesis, wound healing, immune response, as well as in pathologies, especially in cancer growth and metastasis. On the other hand, cell motility is used for cell screening and sorting, and for the design of bio-active surfaces. However, a general understanding of the underlying mechanisms and a prediction of the responses of cells to changes in the environment or external stimuli has not been achieved to date.

Predictive computational modeling will be helpful in this respect, however is a very complex task. It is commonly accepted that the basic processes involved in substrate-based cell motility are actin protrusion via polymerization at the cell’s front (also called the leading edge), the intermittent formation of adhesion sites for the cell to transfer momentum to the substrate, and the detachment of adhesion and possibly myosin-driven contraction at the cell’s rear [1], [2]. While the cell’s protrusion is in itself a complex process [3], [4], on a coarse scale - on the scale of the cell - it can be successfully modeled by the level set [5], [6] or the phase field [7]–[9] methods that track the cell’s boundary (i.e. the membrane) in a self-consistent way.

For reasons of simplicity, however, in most models the dynamics of cell adhesion and its interplay with the substrate properties - like substrate adhesiveness or stiffness - are neglected and the whole complexity of this process is reduced to the level of a simple viscous friction between the crawling cell and the substrate [7], [10], [11]. While this is an acceptable approximation for rapidly moving cells like the often studied keratocytes on substrates with moderate adhesive strength [11], it is commonly recognized that the dynamics of adhesion sites and substrate compliance can strongly affect the shapes, the internal organization, and the overall mode of cell movement. A few exceptions to this simplification are e.g. Ref. [9], where discrete stochastic adhesion sites where introduced while the friction was described by a spatially uniform drag force proportional to the velocity of the cell, and Ref. [6], where the dynamics of the integrin density - the membrane-embedded proteins establishing the link between the substrate and the actin cytoskeleton inside the cell - was modeled explicitly, but the overall effect of adhesion was still an effective friction.

Even less established are the effects of substrate stiffness, which so far have been accounted for only in highly simplified mechanistic models [12], [13], and on the level of force-velocity relations. Due to the complexity of the cellular adhesion mechanism [14], it is obvious that specific questions like mechano-sensitivity [15], or complex motility modes like stick-slip motion occurring e.g. for filopodia [16], can not be understood by the usual approximations, i.e. a simple friction law and neglecting substrate stiffness. Other important phenomena related to adhesion are the effects of artificially designed spatially selective adhesion patterns, recently studied experimentally for both spreading [17] and motile cells [18], as well as the guidance of cell movement by the rigidity of the substrate [19], the so-called durotaxis.

In order to elucidate the effects of adhesion and substrate properties in the modeling of cell movement, here we generalize our model for motile cell fragments proposed previously in Ref. [8]. This model, based on a phase-field approach coupled to the averaged actin orientation (or polarization) field, is significantly extended to account for explicit dynamics of the adhesion site formation and an averaged deformation of the substrate. To reduce the computational complexity, while the density of adhesion sites is spatially resolved, the substrate is treated as an effective (visco-)elastic spring. This simplification has been done in order to extract the generic features of the adhesion dynamics and the effects of substrate stiffness, as well as to make analytic calculations - in addition to the computational modeling - possible, in order to obtain better insight in how adhesion affects cell motion. Despite all the simplifications, we were able to reproduce key experimental observations, such as transitions between steady and stick-slip motion as a function of protrusion rate, adhesiveness and stiffness. In addition we make testable predictions for the locomotion of eukaryotic cells on selectively patterned adhesive substrates and analyze the response of cells to a step in the adhesiveness or substrate stiffness. Both situations have been recently studied experimentally [18], [20].

## Results

### Description of the Model

In Ref. [8], we developed a two-component physical model that was able to account for the key phenomenology of moving cells: a discontinuous onset of motion [21], a broad diversity of cell shapes spanning from crescent-like keratocyte shapes to more fan-like fibroblast shapes [22], as well as shape oscillations.

**Figure 1:**
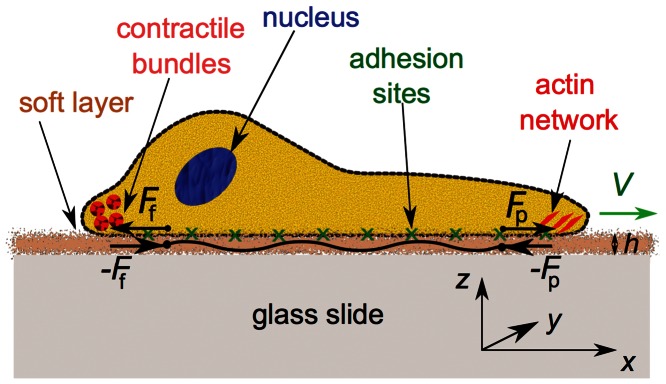
Schematics of the model. The soft (visco-)elastic adhesive layer of thickness *h* is sandwiched between the much less deformable substrate (e.g. a glass slide or PDMS) and the cell, here assumed to be moving in positive *x*-direction with speed *V*. The cell exerts a dipolar force in the layer, illustrated schematically by the propulsion force *F_p_* balanced by the friction force *F_f_*. Correspondingly, a pair of forces of opposite polarity is applied to the cell. The induced deformation in the soft layer is modeled by the extension of an effective spring, with effective spring constant *G*.

The main ingredients of the model are two continuum fields. One describes the two-dimensional cell boundary (phase field) and a second one models, in a simplified way, the dynamics of the actin cytoskeleton. Both equations are coupled, inspired by the relevant biological processes: (i) The polymerization of the F-actin network predominantly occurs near the cell’s boundary. This is due to Wiskott-Aldrich syndrome proteins (WASP) nucleating new branches of actin filaments by activating the Arp2/3 complex located near the cell membrane [4]. (ii) Through nascent adhesive contacts (formed by integrin complexes) the polymerizing actin network, possibly with the assistance of myosin molecular motors [23], is able to transfer momentum to the substrate and to push the boundary forward. Besides these two main ingredients, motor contraction and overall area conservation of the cell are included. Finally, to model keratocytes, an additional symmetry breaking term was added that mimics the effect of myosin motor-induced bundling of actin filaments at the rear, cf. [21]. The present work significantly extends the model to account (i) for an explicit adhesion dynamics, the adhesion strength controlling the propulsion force exerted by the cell. (ii) The adhesion dynamics is self-consistently coupled to the deformation of the substrate the cell crawls on, see Fig. 1 for illustration.

To efficiently track the cell’s interface, as described earlier [8], we introduce an auxiliary phase field 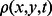, separating the interior of the cell (where 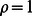) from the exterior (where 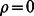) and varying smoothly in between. The diffuse interface is interpreted as the location of the cell’s membrane. The second field, 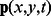, describes the averaged orientation field of the actin filament network. For these two fields the following simple dynamic equations are proposed, cf. [8]:
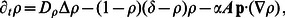
(1)

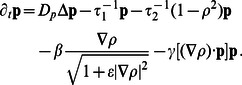
(2)


The first term in Eq. (1), 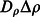, has two meanings: first, it characterizes the width of the phase-field interface. In addition, it describes, on a simplified level, the ratio of the membrane surface tension to the cell’s friction with the substrate [7]. The second term is the variational derivative of a model “free energy”, 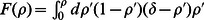, which has a double-well form. For 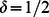 the depths of the two potential wells are equal, and hence none of the two “phases”, 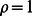 (the cell) and 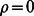 (the outside), is preferred - a situation representing a stationary cell. The last term in Eq. (1) describes the advection of the cell’s interface due to actin polymerization with the propulsion parameter 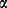. It results in a preference of either 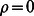, in which case the cell locally retracts, or of 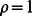, when the cell locally advances. In addition to propulsion due to actin polymerization other possible mechanisms of force-generation are discussed in the context of cell motility, e.g. the combined activity of filament treadmilling and motors [24], [25] or of retrograde actin flow and contractile forces generated by stress fibers [23]. While different in detail, a phenomenological description of this mechanism will be similar to that presented in Eqs. (1,2).

Equation (1) is supplemented by the global constraint
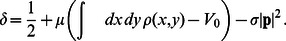
(3)


The two additional contributions in Eq. (3) describe overall area conservation (to preserve the size 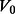 of the cell, with a stiffness of the constraint 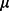) and the actin contraction by myosin motors due to active stresses (of strength 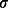), see [8], [26] for details. The parameter 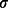 is involved in the control of the cell’s shape: cells assume fan-like (fibroblast) shapes for small values of 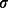, and crescent-like (keratocyte) shapes for larger values, see the Methods section.

Equation (2) models, in a simple way that is inspired by more advanced theories [26], [27] the dynamics of the actin cytoskeleton on the level of its mean orientation. The terms on the r.h.s. describe, in the order of appearance, stiffness/diffusion of the orientation field, degradation of orientation (e.g. by depolymerization, of rate 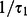), suppression of orientation outside the cell (with rate 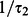), generation of new filaments (with rate 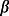) by actin polymerization at the location of the membrane, and finally a symmetry-breaking term mimicking the effect of motor-induced bundle formation at the cell’s rear (see also below).

For a more detailed discussion of the basic model, we refer to Ref. [8]. We would however like to make two remarks: first, we introduced a saturation of the growth term of actin filaments at the membrane, to prevent excessive creation of filaments for very steep 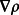 and to suppress this non-physical instability; the parameter 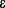 sets the maximum value of the filament growth rate to 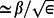. Second, concerning the last term in Eq. (2) that was introduced in Ref. [8] to model keratocyte cells: for keratocytes, and also their fragments, it is known [21], [22] that myosin motors induce an anti-parallel actin bundle at the cell’s rear, apparently to stabilize the cell’s polarized shape. In terms of the vector 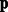, this means that polar orientation is suppressed at the rear, as implemented by the term 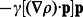. Note that this term, breaking the 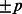-symmetry, favors polarized motile cells. However, our subsequent studies revealed that it is possible to obtain self-sustained cell motion even without this term, namely for large values of the parameter 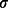 characterizing the overall contraction of the actin network by myosin motors. In this case, the self-sustained motion is the outcome of the shape deformations of the moving cell. This point is discussed in more detail in the Methods section. Within the model given by Eqs. (1–3), there are, therefore, two possibilities for further simplification: one could, in principle, neglect either the last term in Eq. (2) or the last term in Eq. (3) and still obtain moving states.

Let us now discuss the extension of the model towards adhesion and substrate dynamics. First, note that in Ref. [8] adhesion was tacitly assumed to allow the filaments to push against the membrane - i.e. it was acting like a *homogeneous* friction, the usually applied approximation as discussed in the introduction. In contrast, here we have written for the last term in Eq. (1), describing the actin-based propulsion. In principle, the propulsion strength will be a certain function 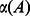. Indeed, it is known that the cell’s crawling velocity is a non-monotonous function of adhesion strength [28], first increasing, reaching a maximum and then decreasing again for too sticky substrates. We here assume 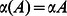 for simplicity. Thus, the propulsion term becomes 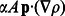, i.e. the propulsion is now explicitly dependent on the number of adhesive contacts, 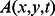. The adhesion contacts describe integrin complexes that are engaged to both the substrate (where ligands like fibronectin or RGD [Arginine-Glycine-Aspartic acid] are assumed to be present) and the cytoskeleton via recruitment of further proteins like zyxin, talin, or vinculin [23], [29]. Note that the parameter 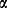 contains properties of the adhesive contacts, for example, the typical force that can be transmitted to the substrate by a single bond.

We now specify the dynamics of the adhesive contacts, and suggest the following reaction-diffusion type equation (a somewhat similar approach was adopted in the context of stick-slip friction in ultra-thin films in Ref. [30]). We would like to stress that this dynamics is highly simplified, as discussed in more detail below. Here we take into account only the most basic features needed to model complex cellular dynamics and substrate dependence. The proposed dynamics couples the number of adhesive contacts 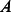, the orientation of the actin cytoskeleton 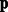, the substrate deformation 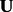, and the shape dynamics described by the phase field 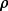:
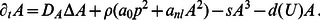
(4)


Here 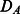 is a (small) diffusion coefficient introduced for spatial regularization of the adhesion site distribution. The following two terms describe the attachment dynamics, and the last two terms model excluded volume interaction and detachment of adhesive contacts, respectively. The term proportional to 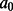 describes the attachment of adhesion sites inside the cell. As effective adhesion forms only if actin is present and independently on the actin network orientation [23], we introduced a direct proportionality to 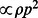. Furthermore, it is known that an already formed adhesion complex favors the formation of other adhesive contacts in the surrounding. This has several reasons: first, due to an already formed contact, the membrane’s fluctuations are locally reduced [31]. Second, the exterior of the cell membrane is decorated by a thin polymer layer (the so-called glycocalyx) that is thicker than the size of the ligand-receptor bond (integrin-fibronectin). Hence, engaged bonds deform the membrane, and, therefore, effectively attract each other, as an aggregation reduces the elastic energy of the membrane [32], [33]. To mimic the nonlinear (multi-body) attachment due to such collective effects, we introduced the term proportional to 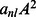. The term 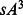 models an excluded volume interaction, leading to a local saturation of the adhesion site concentration.

The last term in Eq. (4), 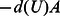, introduces a coupling to an averaged substrate deformation 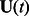. As shown in the sketch of the geometry in Fig. 1, we do not resolve the substrate deformation locally, but model it as and effective spring that has a certain extension 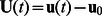 from its equilibrium value 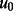, in the absence of the cell. If the absolute value 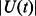 exceeds a critical value 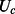, the adhesion contacts will break. For the deformation-dependent breakage rate we state the following computationally convenient form:
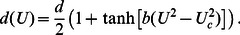
(5)


For high values of 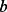, 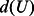 is a step-like function, i.e. zero for 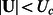 and equal to the detachment rate 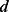 for 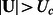.

Let us briefly discuss additional properties of the adhesion dynamics, that could be incorporated in a future, more complete description. The formation and rupture of the adhesive contacts displays many subtleties, some of them still under debate or not yet completely understood. Firstly, the processes are stochastic in nature, which we neglected in our mean-field description. Secondly, the growth of an already formed adhesive contact (and, correspondingly, its maturation) depends on the stresses inferred by both the substrate and actin network. These issues have been addressed in several studies [34], [35], although not on the level of an entire cell. Since we do not resolve local stresses in the cytoskeleton, the substrate effects are included in a highly simplified fashion. Another class of subtleties is related to the molecular scale: the molecular complexity is manifested by the so-called catch bond mechanism, i.e. an allosteric change in the integrin structure under external force, altering its binding affinity. Another molecular consequence is the opening of cryptic (hidden under normal conditions) sites under the applied stress, allowing the recruitment of helper proteins that in turn amplify the recruitment of actin. Hence adhesion in general has, depending on the relevant time scales, a feedback on the actin dynamics [29], [36]. Nevertheless, our simple model of the adhesion dynamics is qualitatively correct for rapidly moving cells like keratocytes, as well as for two generic aspects of cell migration: the occurrence of stick-slip motion and navigation on patterned substrates.

For the subsequent studies, it should be noted that all attachment and detachment rates (i.e. 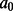, 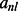 and 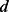) are *effective* parameters including both characteristics of the adhesion complex and its formation *and* characteristics of the preparation conditions of the substrate. For example, varying the concentration of fibronectins on the substrate effectively changes all these parameters. Hence, a spatial modulation of the number of adhesion ligands present on the substrate, cf. the experiments in Ref. [18], will affect the overall dynamics of adhesion. This aspect will be studied in detail in the section **Patterned adhesiveness**.

Finally, we specify a dynamic equation for the extension 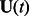 of the spring modeling the overall substrate deformation:
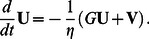
(6)


Here 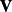 is the velocity of the cell’s center of mass, 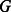 is an effective spring constant and 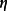 an effective viscous friction coefficient modeling the dissipation mechanisms in the adhesive layer. This equation can be motivated as follows, cf. also Fig. 1: Consider a thin adhesive layer, e.g. a layer of extracellular matrix (ECM) or fibronectin, covering a significantly more rigid glass slide or PDMS (polydimethylsiloxane) substrate. This layer of thickness 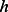 is sandwiched between the cell at position 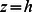 and the much stiffer substrate at 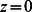. After integrating the force balance equation 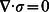 in 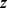-direction, one obtains in leading order in 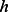:
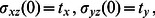
(7)where 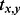 are the traction forces on the top of the layer at 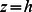. We further assume the layer to be rigidly fixed to the substrate (i.e. the displacements 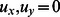 at 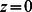), and a planar shear 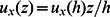. Now we have to specify the constitutive relation of the layer. At long times, the response will be elastic. However, at short times there can be (e.g. viscous or breakage-induced) dissipation in general. Hence we use a Voigt-Kelvin-type viscoelastic solid model for the layer, and write



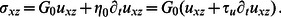
(8)Now, as we do not want to resolve the displacement locally, we need an integrated quantity describing the stresses/forces induced by the cell.

Since the cell does not exert a net force on the substrate, the total traction, 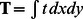, is zero and can not be used for this purpose. However, the traction dipole moment 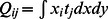 is nonzero (compare e.g. [12]). We assume here, for simplicity, that the traction force 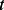 associated with the motility is parallel to the cell’s migration velocity, 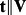. The traction pattern for keratocytes is not yet understood in detail. There is typically high traction at the sides [36], related to the actomyosin bundles spanning at the rear to both sides. Nevertheless, the traction relevant for motion is the dipole in the direction of motion.

The traction can then be written as 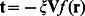, where 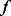 is a localized, dimensionless function having zero average, 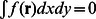, but a finite dipolar contribution. The coefficient 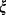 has units of friction per area, 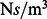. Note that there is also a velocity-independent contribution to the force dipole and hence to the traction force: immobile cells exert forces on the substrate as well, but this part does not affect the dynamics - namely, it can be absorbed in 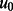 and leads to a renormalization of the parameter 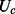 - and can be discarded here.

Finally, from the 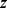-integrated force balance, Eq. (7), and accounting for the constitutive relation, we get by multiplication by 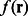 and integration over the area of the cell
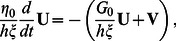
(9)where 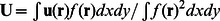. The vector 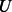, having units of length, is a measure for the overall deformation imposed by the cell on the substrate. Defining a renormalized viscous coefficient 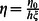, and a renormalized stiffness 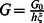, we obtain Eq. (6). Note that a relaxation time for the adhesive layer is defined by 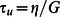.

### Steady vs. Stick-slip Motion

There are many realizations of unsteady motion in the context of cellular motility. It has been found that lamellipodia can show periodic contractions that depend on the adhesiveness of the substrate [38], later on interpreted by a stick-slip model [39]. Stochastic modeling of the adhesion dynamics under applied forces and flows also predicted the occurrence of stick-slip events [35]. Experimentally, stick-slip-like motion has been found in parts of the lamellipodium, e.g. in osteosarcoma cells [40], as well as in filopodia [16]. Finally, transitions from steady motion to stick-slip and to arrest of motion have been observed in human glioma cells cultured on extracellular matrix (ECM) [41]. We will now investigate the consequences and generic features of the adhesion dynamics and substrate properties in the proposed model, and analyze in detail the occurring stick-slip behavior.

**Figure 2:**
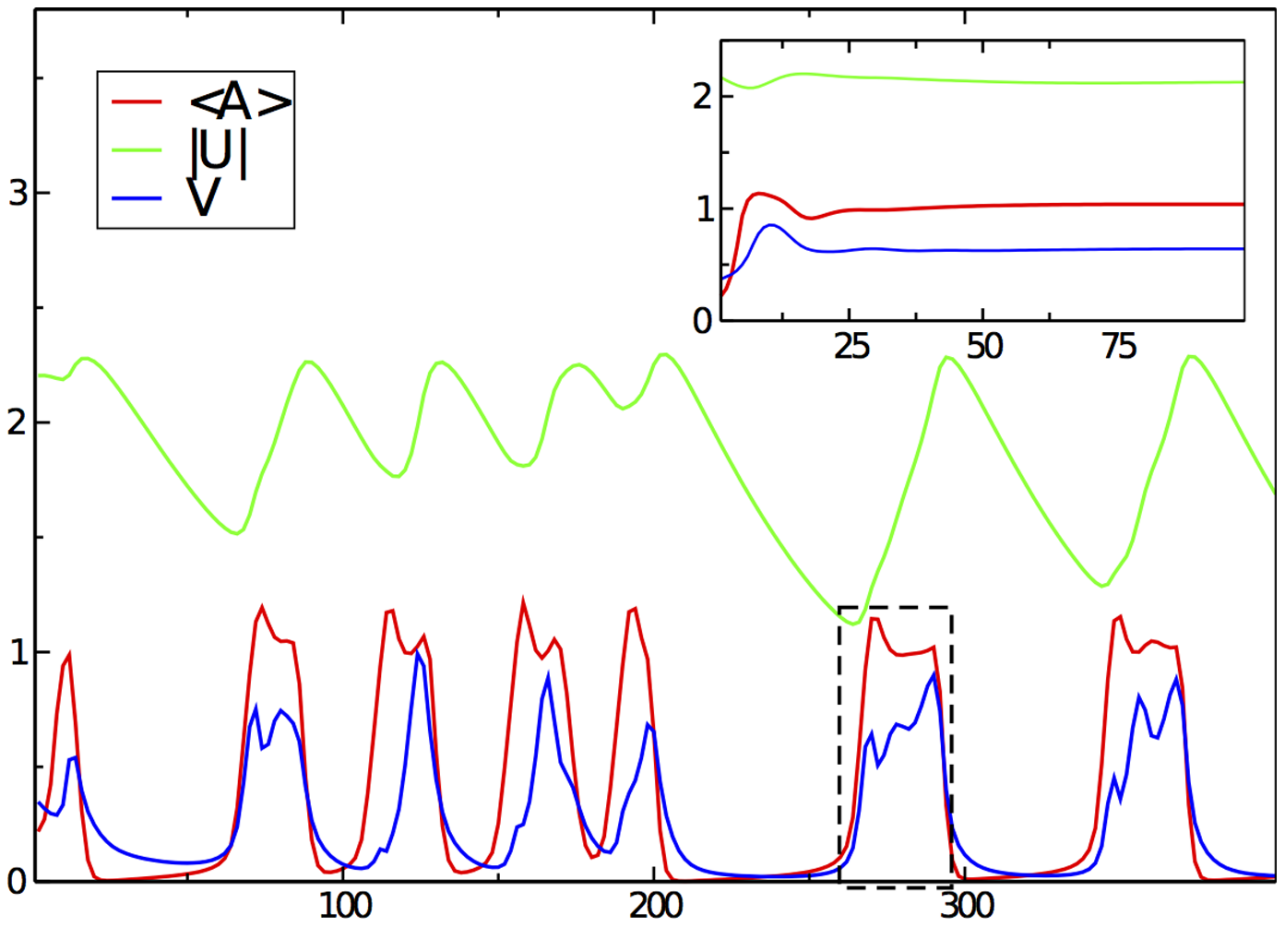
Dynamic modes of motion. The main figure shows a typical stick-slip motion. The inset displays steady-state motion for high substrate stiffness and otherwise same parameters and initial conditions. The integrated adhesion density 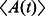 is shown in red and the velocity 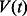 of the cell in blue. The absolute value of the extension (in the direction of motion) of the effective spring modeling the substrate, 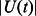, is shown in green. The dashed box is the time window where Figure 2 displays the spatially resolved dynamics. Parameters: 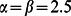, 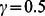, 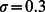, 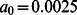, 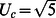, 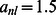, 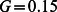; initial area of the cell 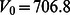 (initial radius 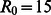). Other parameter as given in Table 1.

We first investigated whether the generalization still comprises the phenomenology of the model described previously. This can be tested by choosing a high value for the substrate stiffness, since then the spring extension will not play a significant role. Indeed, after a transient, the system acquires a steadily moving state with the number of adhesion sites 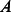 and the substrate spring extension 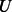 reaching time-independent values as shown in the inset of Fig. 2, see also Movie S1. This is exactly what the model developed in Ref. [8] already predicted, but with the propulsion parameter 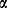 (proportional to the actin polymerization rate and substrate adhesiveness) replaced by an effective propulsion strength 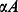. Keeping the substrate stiffness 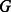 high and decreasing the value of 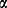 finally leads to a termination of motion, again in accordance with the previous results: in this case, the polymerization force is not high enough to sustain the polarized moving state and the cell will stop and acquire a radially symmetric shape. Finally, in the steady-state regime the cell’s speed is almost independent of the substrate stiffness 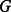 and linear attachment rate 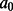. Note that in this regime, the displacement of the spring 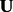, modeling the substrate deformation, is below the critical extension for bond breaking 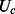. Hence, the number of adhesion sites is governed by the attachment and excluded volume effects.

Having this established, we can study the model in a broader range of parameters. One would expect the occurrence of a stick-slip regime by the following generic mechanism: When the adhesion sites are forming, the cell speeds up. Since the cell, adherent to the substrate, exerts dipolar forces on the substrate, the substrate deformation increases in its absolute value, and for low enough stiffness 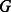 to an extent that the critical value 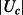 is reached and the adhesive contacts rapidly break. However, the cell has still to slow down and adjust its shape to the new conditions. If the substrate relaxes, new adhesion sites are allowed to form again and the cycle restarts. Figure 2 shows the dynamics of the cell’s velocity 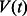, the integrated density of adhesive contacts 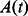, and the substrate extension 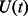 for typical parameters in the intermediate stick-slip regime. Note that due to the coupling to the shape dynamics, the stick-slip oscillations are not strictly periodic in general, although this can be the case for some parameters (cf. Movie S2). The average period of the stick-slip cycle increases with a decrease of the attachment rate 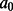, roughly linearly, and the effective stiffness 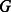.

**Figure 3:**
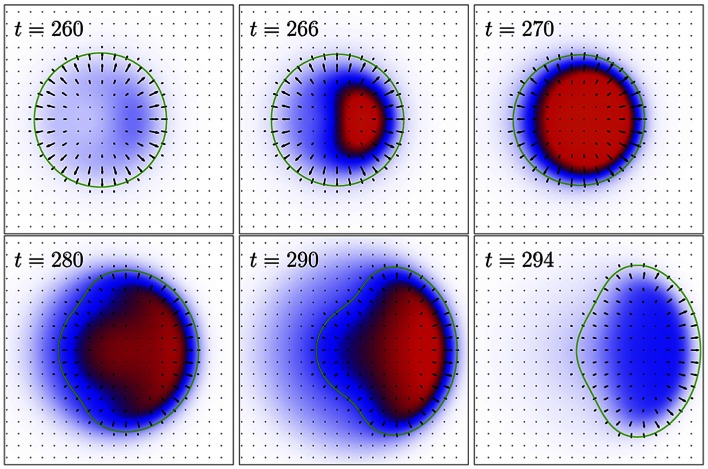
Spatially resolved dynamics during a stick-slip cycle. The cell’s boundary is given by the green curves. The arrows display the local averaged actin filament orientation. The density of adhesion sites is color coded (with white color corresponding to 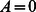, blue to 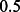 and red to 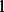). The direction of motion is to the right. The time window is the one marked by the dashed box in Figure 2. Between 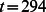 and the next attachment event, the cell will relax to an almost round state, similar to the one displayed for 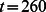. Parameters as in Fig. 2.

Figure 2 showed only averaged quantities. The shape deformations and the local dynamics of the adhesive contacts are depicted in Fig. 3. It illustrates the shape of the cell (represented by the green curve), the local actin orientation (indicated as black arrows) and the local distribution of adhesive contacts during the stick-slip cycle highlighted by the dashed box in Fig. 2. The concentration of adhesive contacts 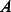 is color coded, with white corresponding to 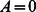, blue to 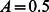 and red to 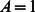 [which is the maximum value, as can be tuned by the excluded volume term in Eq. (4)]. The respective times are added in the panels. At first, there are practically no adhesive contacts and the cell is almost round. In the next panel, adhesion contacts form close to what becomes the leading edge of the cell (the cell is moving to the right). This is due to the fact that the actin concentration proportional to 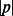 is slightly higher there, a remnant from the last cycle. Then adhesion site formation spreads over the entire cell for the given parameters. Note that there is a range of model parameters, as shown in Movie S2, where adhesive contacts form only close to the front. In our simple model this is determined mainly by the time scales of adhesion and substrate dynamics and the value of the diffusion coefficient 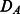.

Since the distribution of adhesive contacts is not symmetric - they formed earlier close to the leading edge - the cell is able to polarize and starts to move again, until the substrate displacement generated by the cell reaches the critical value 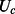 and adhesion breaks down. As the propulsion force decreases, the cell slows down and becomes more round, i.e. depolarizes, cf. the last panel.

**Figure 4:**
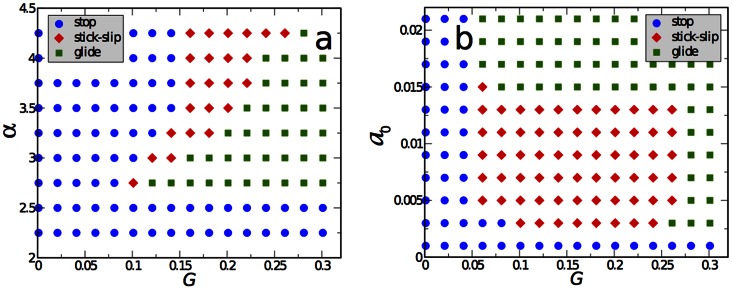
Phase diagrams. a) The different modes of motility in the plane of propulsion parameter 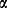 vs. substrate stiffness 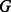 for 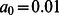 and 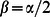. b) The different modes of motility in the plane of rate of adhesion formation 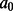 vs. substrate stiffness 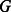 for 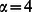, 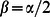. For both parts, parameters where a cell stops after an initial perturbation are marked by blue circles. If stick-slip motion is persistent this is marked by red diamonds and if the cell acquires a steadily moving state (continuous gliding), by a green square. Other parameters as in Fig. 2.

By performing large scale parameter sweeps, we obtain dynamic “phase” diagrams for the different modes of cell motion, as shown in Fig. 4. In part a) we varied both the substrate stiffness 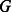 and the propulsion parameter 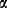, which is related to the actin polymerization rate. In agreement with the above discussion, for high enough substrate stiffness and propulsion, the system displays steady-state motion with a fixed shape, reminiscent to keratocyte-like motion. Decreasing the stiffness, at intermediate values a region of persistent stick-slip motion appears, while for small stiffness the cell is unable to move and stops. On the other hand, upon a decrease of the propulsion parameter 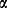, the size of the stick-slip region shrinks until the cell is completely unable to move below a certain critical 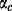.

Figure 4b displays a similar plot, where the rate of adhesion formation 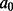 and the substrate stiffness are varied. Similar as for the propulsion parameter 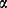, there is a threshold value 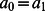 for the adhesion rate below which the cell is not able to move - adhesion is just to weak to transfer momentum. Increasing 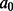, one obtains a steady motion for high substrate stiffness, stick-slip motion for intermediate values and no motion for small stiffness. The width of the stick-slip region is fairly independent of 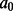, until it abruptly ceases to exist for a second threshold value 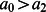; above this threshold the formation of adhesion sites dominates detachment, resulting in steady gliding motion.

Similar diagrams as shown in Figure 4 can be in principle obtained by varying other relevant parameters. The above study already demonstrated that the type of cell motion and the cell shape are governed by the interplay of (i) the motility machinery of the cell, exemplified here by the propulsion parameter 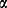 which is proportional to the actin polymerization rate, (ii) the adhesion dynamics, exemplified here by the adhesion rate 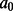 depending also on the substrate’s surface preparation, as well as (iii) the elastic properties of the substrate/the adhesive layer, i.e. the stiffness 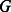 and the relaxation time 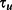.

Finally, one should note that the stick-slip motion of cells adherent to a substrate is somewhat different from a classical view of stick-slip motion [42], [43], illustrated by a brick pulled by a spring on an adhesive layer: there no motion occurs while the brick is stuck, while the brick moves upon a slippage event after bond breaking. In contrast, for cells crawling on a substrate, the motion is mostly generated upon adhesive contact, where the cell polarizes and momentum from actin polymerization can be effectively transferred to the substrate, while the shape accommodation after the bond breaking has only a minor contribution to motion. Hence instead of stick-slip cycle, a better name for this process occurring in crawling cells would be propulsion-relaxation cycle, cf. also Fig. 3.

### Reduced Description of the Stick-slip Cycle

To obtain a better understanding of the stick-slip motion, we will reduce the full model, Eqs. (1)–(6) to two effective ordinary differential equations (ODEs): one for the area-integrated density of adhesive contacts, 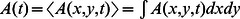, and the second for the extension 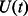 of the spring, modeling the overall substrate deformation.

For this purpose we first neglect all diffusion terms. We also disregard the effects of the orientation field 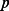 and assume that the cell is already polarized and able to move. We can then assume a one-dimensional motion. In this approximation the phase field equation yields a relation between the cell’s velocity 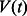 and the mean polarization, 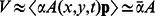, where 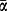 is a numerical factor. For a more elaborate description see Eq. (16) in the Methods section. Similarly we obtain 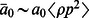 and arrive at:
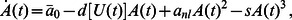


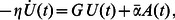
(10)where the deformation-dependent detachment rate is still given by Eq. (5).

**Figure 5:**
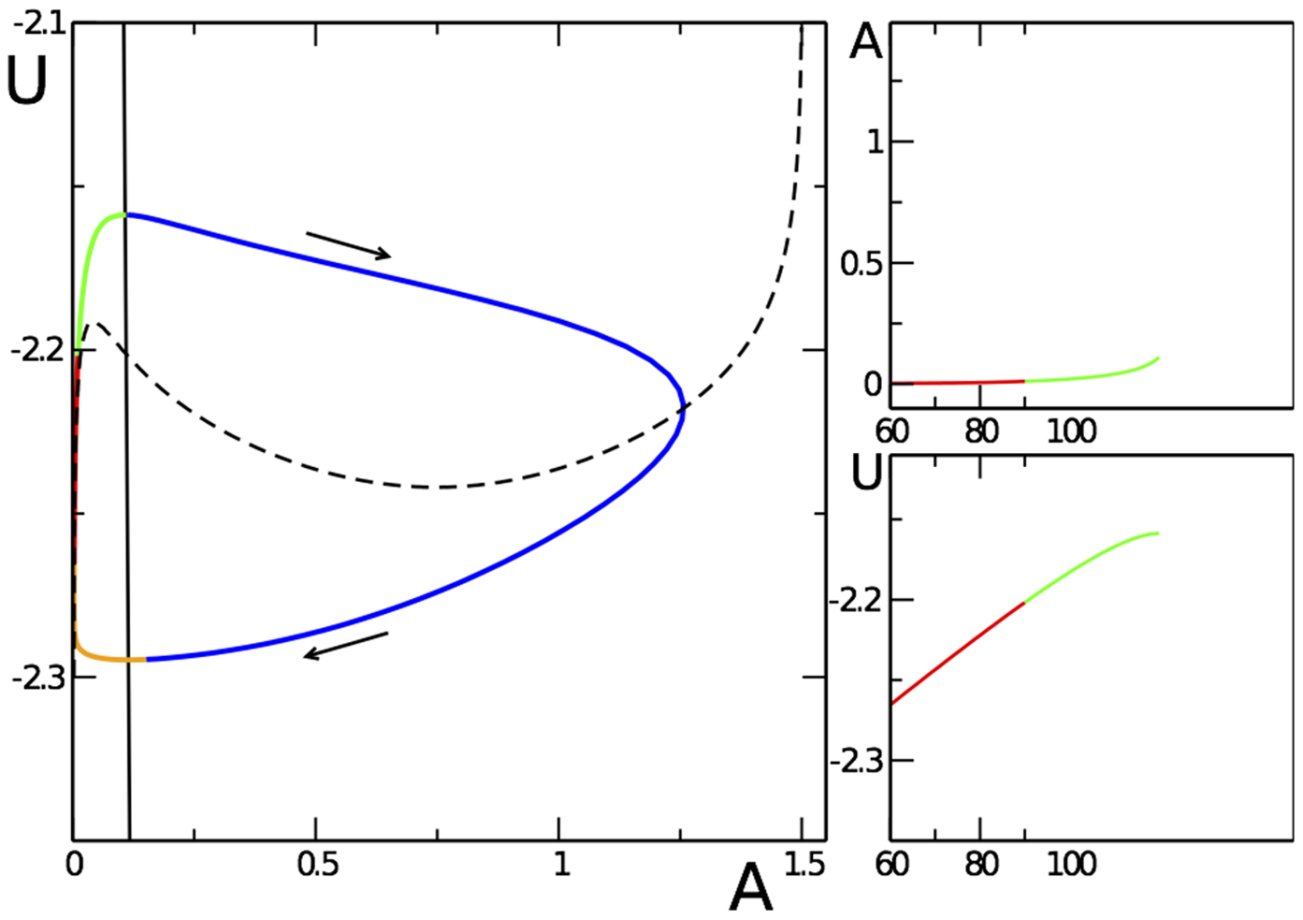
Stick-slip cycle in the reduced ODE model. The main plot shows the nullclines 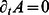 (dashed line) and 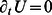 (solid line) and the limit cycle (with each quarter period marked in a different color) obtained by numerical integration of Eqs. (10). Starting at the upper part of the blue trajectory, the cell increasingly adheres and 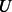 becomes more negative as the cell exerts more and more force on the substrate. When 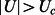, adhesive contacts break and 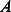 rapidly decreases (lower part of the blue trajectory) until the dynamics reaches the 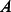-nullcline (red branch). There 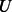 relaxes while 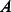 almost stays zero, but effectively slowly grows as at small values of 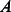 the cell slows down and adhesion can restart (green trajectory) followed again by rapid attachment (blue trajectory). Parameters: 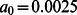, 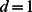, 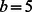, 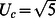, 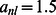, 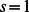, 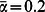, 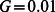, 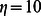. The two panels on the right show 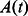 and 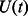 for one period of the stick-slip cycle.

Eqs. (10) is a second-order system of equations that can be easily integrated numerically. In addition, the stick-slip motion can be understood qualitatively from the analysis of nullclines 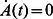 and 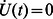. Both nullclines (solid black line for 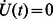, dashed black curve for 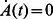) and the actual trajectory - obtained by solving Eqs. (10) numerically - are shown in Fig. 5. The stick-slip motion corresponds to a periodic trajectory (limit cycle) encircling the unstable fixed point at the intersection of both nullclines. We can also discuss the dependence on parameters by this method: Increasing the stiffness of the substrate 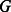 (or decreasing the propulsion 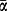) results in a decrease of the slope of the nullcline 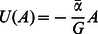 (solid line). At some critical value of 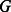 this nullcline will have additional intersections with the second nullcline (the dashed line), resulting in the disappearance of the limit cycle and the creation of a new stable fixed point corresponding to the steady motion of the cell. The same happens - when starting from the stick-slip regime - upon decrease of 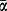, cf. the transition from stick-slip to steady motion upon lowering 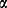 in Fig. 4a. Finally, upon an increase in 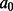, the maximum in the dashed nullcline for 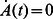 decreases and is shifted to higher 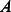 values. As soon as the fixed point, i.e. the intersection of the nullclines, is no longer on the descending branch of the dashed curve, steady motion occurs again, cf. the transition from stick-slip to steady motion upon increase in 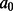 displayed in Fig. 4b.

In addition, Figure 5 displays 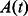 and 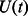 obtained from Eqs. (10) by numerical integration. The color code splits the period in four. One can see that the attachment-detachment event [cf. the blue peak in 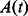] is very rapid. The curves are very similar to ones obtained by the full model, cf. Fig. 2. However, due to the complete omission of the shape and polarization dynamics - as well as of the subcritical onset of motion, cf. the Methods section - the cycles are perfectly periodic in the simple model.

### Patterned Adhesiveness and Patterned Substrate Stiffness

The extended model also allows to study the effects of modulated substrate properties. Note that from the experimental perspective, it is much easier to engineer and pattern the substrate properties and to study the respective response of the cells than to modify the intertwined biochemical processes inside the cell. We focus on two generic geometries: (i) a step in substrate property (adhesiveness, stiffness) and (ii) a periodic modulation of substrate property, exemplified by an periodic array of adhesive stripes, similar to the experiments in Ref. [18]. Experimentally, the adhesiveness can be changed by varying the density of integrin ligands on the substrate; for example, using photolithography techniques based on cleavage of the PEG (polyethylen glycole) layer upon UV exposure, nowadays almost any adhesive pattern can be engineered [44]. On the other hand, the substrate stiffness can be varied, either in a gradient fashion [19] or step-like using soft lithography fabricated microposts [20].

#### Steps

**Figure 6:**
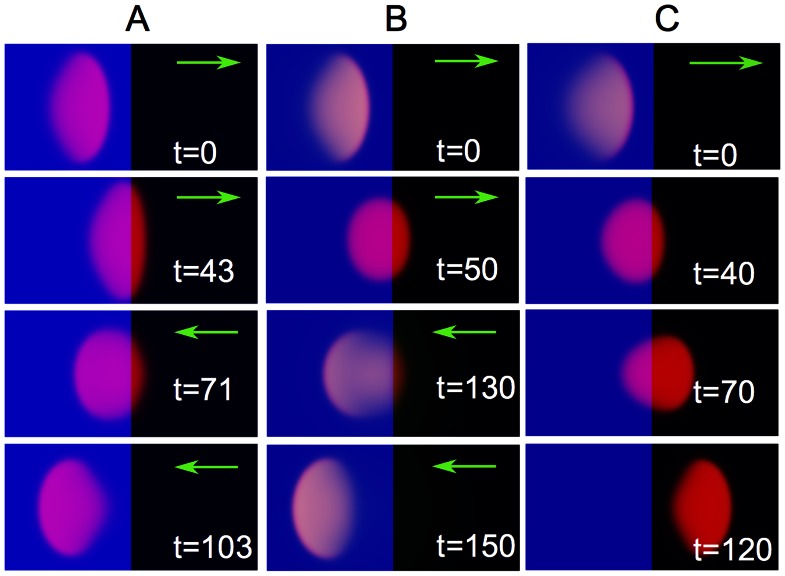
Response to step-like changes in substrate properties. A) Motion of a cell on a substrate where the adhesive strength is modulated by a step in the rate of adhesion formation 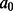, corresponding to a varying density of adhesive ligands (e.g. different surface coverages of fibronectin). The blue region has 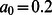, the black one 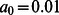, substrate stiffness is 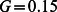. B,C) Motion of a cell on a substrate where the substrate stiffness exhibits a step (the blue region is a rigid substrate with stiffness 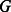, the black one is much softer, 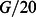). B) The cell bounces off the step for 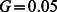, 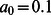 within the entire cell and 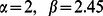. C) The cell overcomes the step and continues in the same direction for 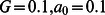, 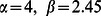. All other parameters as in Fig. 4.

The snapshots in Fig. 6A illustrate cell motion on a substrate with a step in the adhesion strength, modeled by a step-like spatial variation of the rate of adhesion formation 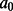. The blue area corresponds to high adhesiveness, the dark one to low adhesiveness. One sees that the cell is capable of navigating on patterned substrates: it bounces off the low-adhesion region back to the region with higher adhesion, see Movie S3. This behavior is in qualitative agreement with experiments confining cells in regions of high adhesiveness, cf. e.g. Ref. [44]. Figures 6B,C illustrate cells moving on substrates with variable stiffness, as a simplified example of mechano-sensitivity. In this case the spatially-dependent substrate stiffness is modeled by a step in the effective spring constant 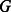. We observed three possible scenarios, depending on the difference in stiffness 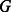, the strength of adhesion 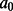, and the value of the propulsion parameter 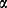. For cells moving on a relatively soft substrate, and for small values of 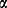 (i.e. for relatively slow moving cells), the cell bounces off the region of low substrate stiffness and chooses to stay on the more rigid substrate, Fig. 6B. On stiffer substrates and for larger values of 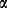, however, the cell can overcome the step in stiffness and continues in the same direction, although with a smaller speed, cf. Fig. 6C. For intermediate values of 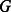 and 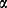 we observed that cells often become trapped at the boundary between the high/low stiffness domains. In contrast, for similar parameters, a cell coming from the softer side became trapped at the boundary between soft/hard substrates and did not reflect back. These results are in qualitative agreement with experiments in Ref. [20]: there a step in substrate stiffness was implemented by regions of differently dimensioned posts on which the cells adhere (while the overall adhesiveness was kept the same). Fibroblast cells preferably stayed in the area of high stiffness, while cells coming from the softer side often rotated to migrate perpendicularly to the stiff substrate [20]. Hence already the simplified model developed here shows that the outcome of a cell’s “collision” with a step in substrate parameters depends quite sensitively on the cell’s shape and speed, as well as on the relative difference in adhesiveness/substrate stiffness.

#### Striped substrates

**Figure 7:**
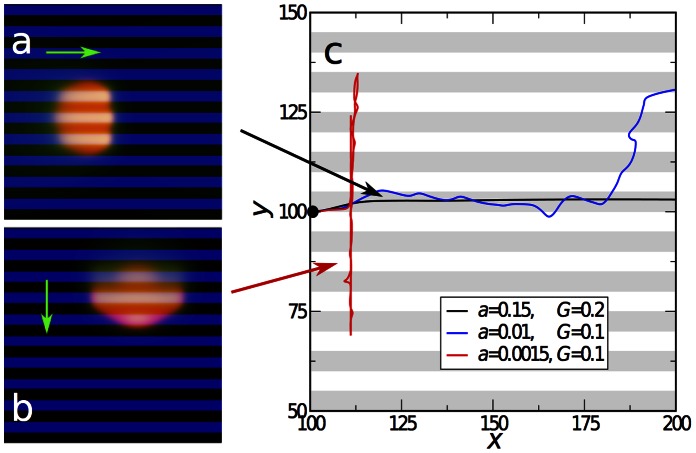
Motion of cells on substrates with alternating stripes of high/low adhesiveness. a) Motion of a cell on a rigid substrate (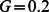) with alternating stripes of high adhesiveness parameter 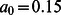 (blue) and no adhesiveness (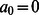 for black stripes). The cell positions itself symmetrically and moves parallel to the stripes in a steady fashion. b) Motion of a cell on a substrate with 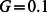, and with alternating stripes of low adhesiveness parameter 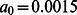 (blue) and 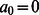 (black). After moving initially along the stripes, the cell turns and moves perpendicular to the stripes in a stick-slip fashion. Parameters are as in Fig. 4 except 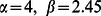. c) Select trajectories of the center of mass of cells moving on stripe-patterned substrates with different values of the adhesion formation rate 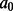 and substrate stiffness 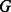. Gray stripes correspond to high adhesiveness regions, white stripes correspond to zero adhesiveness regions (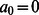). For high 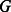 and 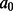, the cell displays persistent and steady motion along the stripes (black curve). For intermediate values the predominant motion is along the stripes with excursions into the perpendicular directions (blue curve). Finally, for low adhesiveness the motion is perpendicular to the stripes with reversals.

We also investigated the motion of cells on striped substrates with alternating high/low adhesiveness. This situation was studied experimentally for keratocytes in Ref. [18], using micro-contact printing of fibronectin for regions of high adhesiveness and of poly-L-lysine-PEG blockcopolymers for practically non-adhesive regions. In the model, again the selective adhesiveness of the substrate is modeled by a spatial modulation of the rate of adhesion complex formation 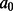. For stripes with large values of 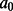 we observed that the cell positions itself symmetrically with respect to the stripes, see Fig. 7a and Movie S4 (i.e. the center-of-mass of the cell drifts to the center of the high adhesiveness stripe) and that the cell moves along the stripes. This behavior agrees well with the experimentally observed one [18]. Remarkably, in faithful agreement with the experiment, the leading edge of the cells exhibits “protrusion bumps” on high-adhesiveness stripes and “lagging bumps” on the stripes of low adhesion.

However, a fundamentally different behavior was obtained for cells moving on striped substrates with lower values of the adhesion parameter 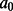, i.e. in the regime where the homogeneous system may display stick-slips. The displayed cell was stimulated to move along the stripes by the initial conditions (the initial polarization was chosen in the direction parallel to stripes), as in the above case. However, after some time the cell slows down, abruptly changes the direction, spreads along the stripe in order to maximize the contacts with the high-adhesiveness region, and begins to move perpendicular to the stripes, cf. Fig. 7b and Movie S5. This motion is associated with stick-slip, where the cell intermittently almost stops along the adhesive stripe building up adhesion strength, and then moves again. We also observed that in this regime the cell may randomly reverse the direction. Thus, our model makes a nontrivial prediction on a new type of motion on patterned low adhesive substrates.

For intermediate values of the adhesion parameter 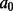 we observed a combination of these two modes of motions: for some time the cell moves along the stripes, then it moves perpendicular, then parallel again, etc. The representative trajectories of the cells are summarized in Fig. 7C.

**Figure 8:**
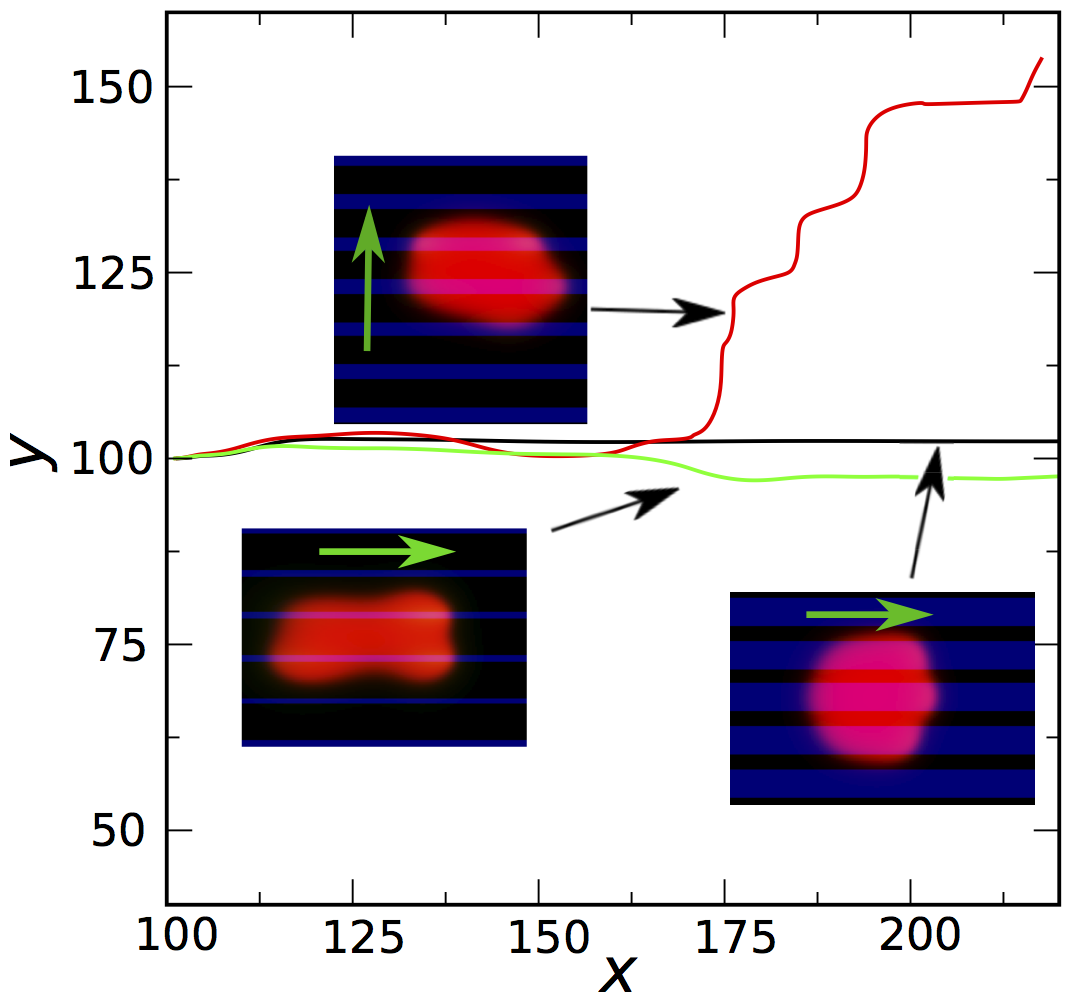
Trajectories of cells on substrates with three different width ratios of adhesive to non-adhesive stripes. In the corresponding snapshots, the adhesive stripes are shown by the blue color and the green arrows indicate the direction of motion. All parameters are as for Fig. 7, except for 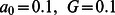 (all parameters are the same in the three cases, only the width ratio changed).

Experimentally, it is rather difficult to modulate the strength of adhesion to a large extend. However, it is relatively simple to vary the relative width of adhesive/non-adhesive stripes, while keeping the period of the pattern fixed [18]. We investigated this situation in our model and observed three different types of motility, see Fig. 8. For large widths of the adhesive stripe, the cell moves along the stripes in agreement with previous simulations. Gradually decreasing the width of the adhesive stripes, we observed an instability: the cells exhibits a kind of “rocking motion”, and eventually turns perpendicular to the stripes. The motion is associated with a random reversal of direction, similar as on the substrates with low value of the adhesion rate 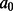 discussed above. Remarkably, for very small widths the reverse trend is observed: the cell stretches in the direction of motion in order to fit between two stripes, and moves along the stripes. These simulations signify that the type of motion is affected by the commensurability between the size of the cell and the period of the modulation, another prediction that deserves experimental investigations.

## Conclusions and Discussion

The sensitivity of cell response on a modulation of the substrate properties has clear implications for the design of bioactive surfaces and test assays for cell screening and sorting. There are increasing experimental efforts to perturb cells externally via the substrate, both on a subcellular level [18] and on larger scales [11], [19], [20], [44], by structuring the substrate, or changing its adhesiveness or stiffness. This calls for modeling efforts where the cell’s shape, internal cytoskeletal and adhesion dynamics, as wells as the substrate compliance are taken into account in a self-consistent, dynamic way. As a first step in this direction, we added a generic dynamics for adhesion and overall substrate deformation to a simple and computationally efficient phase-field model developed in Ref. [8].

The generalized model has been analyzed in detail and displays the following new features: (i) For homogeneous substrates, a transition from steady motion with a fixed shape (reminiscent to keratocyte-like motion) to stick-slip motion with concomitant oscillatory shapes occurs, which we study as a function of actin protrusion rate, adhesion kinetics and effective substrate stiffness. (ii) For substrates with spatially varying adhesiveness we obtain the phenomenology experimentally found in Ref. [18]: On a step profile from high to low adhesiveness, the cell can bounce off. On substrates patterned with alternating high and low adhesiveness stripes, we observed motion along the stripes. In addition the cells were migrating towards the center of the stripe to symmetrize their position with respect to the stripe pattern and they exhibited protruding and lagging bumps, in good qualitative agreement with Ref. [18]. We rationalize this behavior analytically (within the approximation of a spherical cell) and show that the cell’s motion corresponds to an overdamped motion in an effective periodic potential defined by the stripes. Our model also provides nontrivial predictions: while for high adhesion rates we observed an alignment of the direction of motion along the stripes, for lower adhesion rates irregular motion perpendicular to the stripes occurs. Similar trends are found for cells moving on substrates with different width ratios of the adhesive to non-adhesive stripes: for the case of wide adhesive stripes cells move along the stripes, while for narrower adhesive stripes they move perpendicular. The trend reverses for very narrow stripes: the cells tend to fit in between neighboring stripes and move parallel to them. These interesting predictions deserve experimental validation.

Our investigation of the response of cells on a step-like variation of the substrate stiffness also revealed nontrivial behavior. There is an overall trend that cells prefer to stay on the more rigid substrate, as found in many experiments [19], [20]. However, depending on the parameters of our model (especially the propulsion strength, i.e. the actin polymerization rate), the cell can bounce off, become trapped at, or overcome the step in the substrate stiffness. It is evident from the shape changes during these processes that the interplay between shape and adhesion is very important, however a detailed study would need a model with spatially resolved substrate deformations.

While our model demonstrated qualitative agreement with experimental observations on a variety of shapes and responses to external stimuli, and even yielded testable predictions, obviously several extensions are still needed for a more adequate description of the biological system. First, a more realistic treatment of the substrate’s elasticity is needed for detailed studies of the cell’s behavior close to a step in substrate stiffness, as well as for durotaxis [23]. Instead of an effective spring, as proposed here for simplicity, a full two-dimensional linear elasticity model should be used and also the cell’s velocity has to be calculated locally. In such a refined model, adhesive contacts will stretch (and break) only locally, making an analysis much more complicated (imagine locally sticking and slipping regions) but far more realistic. The experiments reported in Ref. [11] might also be modeled by such an extension: there, it was found that on low adhesive substrates keratocytes are almost round and move with erratic protrusions, on substrates with intermediate (optimal, cf. [28]) adhesiveness keratocytes had the usual crescent-like shape and move steadily, while for high adhesiveness they were very irregular, as well as performed erratic motion again. Note that the first transition is somewhat reminiscent of the transition from stick-slip to steady motion in the present model. Finally, such a model extension would allow to obtain explicit traction fields, that could be compared with those experimentally measured for various cell types (cf. Ref. [37] for keratocytes).

A second interesting and important direction is the incorporation of the cell’s intrinsic (visco-)elasticity into the model. First, apart from the elastic deformation and the viscous losses in the adhesive layer, stresses that build up and relax in the cytoskeleton are a second major source for the occurrence of irregular and stick-slip-like motion. Second, such an extension can possibly allow for a more accurate description of the bipedal motion of keratocytes observed in Refs. [45], [46]. In this respect it is interesting to note that our model shows a regime reminiscent of the bipedal (rocking) motion, when the cell moves on adhesive stripes, see Fig. 8. In this case, the reason for the cell’s rocking motion is the inhomogeneous distribution of adhesion sites due to the presence of the stripes. Indeed, for keratocytes adhesion sites are concentrated at the front and mostly at the sides of the cell [11], [37]. Thus, the coupling of adhesion dynamics and cell elasticity may well be responsible for the bipedal motion.

Finally, the main type of cells considered here, keratocytes, move in a very persistent way. In order to identify and separate the effects of the patterned substrate, other possible factors inducing changes in the direction of motion were not implemented. However, for other types of cells, it would be interesting to include stochasticity in polymerization and/or adhesion turnover. In addition, a random anisotropy or heterogeneity of the substrate could lead, depending on its characteristic scale, to abrupt changes in the direction of motion as well.

## Methods

### Numerical Method

Equations (1)-(6) were solved numerically by the quasi-spectral Fourier method in large domains [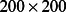 dimensionless units, 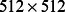 FFT (fast Fourier transform) harmonics used]. The algorithm was implemented on GPUs (graphical processing units) using the NVIDIA CUDA programming language, resulting in an overall speedup of about 50 times compared to CPU. The GPU implementation hence allowed us to explore systematically the system in a wide range of parameters and under different conditions.

### Parameter Estimates

Estimates and typical values for the parameters used in the model are presented in Table 1. As it was explained in detail in Ref. [8], we set the time scale to 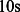 and the length scale to 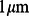 by choosing the parameters of the motility machinery (actin polymerization rate and diffusion) accordingly.

**Table 1:** Parameters.

parameter	value	description
parameters of motility machinery		
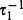	0.1	degradation of 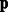 inside cell
		(actin depolymerization rate of 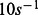 )
		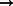 sets time scale to 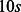
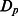	0.2	diffusion/elastic coefficient for P
		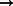 sets length scale to 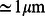
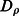	1	stiffness of diffuse interface
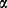	0.5–3	advection of 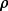 by P
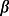	1–2	creation of 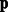 at interface
		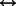 actin polymerization velocity 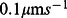
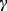	0–2	symmetry breaking due to motors,
		corresponding motor velocity 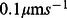
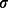	0–2	network contractility by motors
parameters for adhesion turnover and substrate		
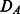	1	diffusion of adhesion sites (AS)
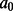	0.001–0.03	linear attachment rate of AS
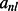	0.5–1.5	collective (nonlinear) attachment rate of AS
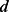	1	detachment rate of AS
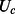	1–5	critical extension to break adhesive contacts
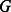	0.01–0.5	substrate stiffness
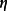	10	dissipation in the adhesive layer

This table shows a summary of the most important parameters of the model. Typical rescaled values used in the numerical solution and their relation to typical values for the “real” system are also given. Additional parameters: stiffness of volume conservation: 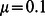; decay rate of 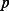 outside the cell: 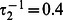; overall area of the cell: 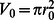 (typically with 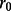 = 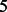
–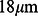); regularization parameter for actin creation: 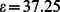; parameter describing the steepness of the detachment transition: 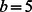; coefficient for nonlinear saturation of adhesion sites: 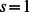.

Typical lateral diffusion coefficients of proteins in a membrane are 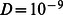-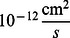 [47]. Hence we can estimate 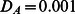
–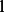 in reduced units. For numerical reasons we use 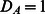, in accordance to Ref. [11]. While the strength of adhesion forces of crawling cells are well established [29], [37], [48], not much is known about the dynamics, i.e. the rates of adhesion contact formation in such a non-equilibrium situation. Nascent adhesions typically form on a time scale of seconds, but the connection to the actin network and the maturation to focal adhesions can take tens of minutes [29]. In modeling efforts for cell motility, cf. Ref. [6], [11], usually rates are adjusted to obtain realistic dynamics on the spatial scale of the cell and on the time scale relevant for motion. For the source term of adhesive contact formation, we chose 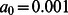
–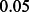. In addition we have the term 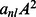, describing collective adhesion, which is chosen to be in the range 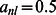
–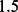 - note that it has different units than 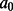 and should be attributed a larger value, due to the quadratic dependence on the adhesion site density 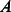. Our detachment kinetics is dominated by the deformation of the substrate, placing the adhesion complexes under stress, and we typically chose 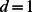 (i.e. rapid breakage of adhesion sites) and 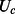 of the order of micron.

Finally, typical traction forces are of the order of 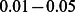 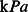 [36]. Together with the typical cell speed, 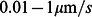, this leads to 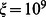-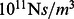. The typical thickness of the adhesive layer plus the typical depth of substrate deformation is of the order of several microns. For soft substrates, 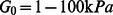, we can therefore estimate 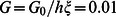
–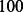. The viscous losses are described by 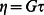, where we assume a relaxation time of 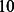
–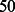 s. We typically use 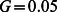
–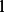 and keep 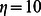 fixed.

### Circular Approximation

In this and the following sections we develop a reduced description for steady cell motility, as well as for the response to a substrate modulation, rationalizing why the cells move along the stripes in case of steady-state motion, cf. Fig. 6a. The main simplification is to assume a fixed round shape of the cell (a somewhat similar approach was used in Ref. [49] in the analysis of a bifurcation to traveling localized spots in a reaction-diffusion system). In spite of this approximation, one should be aware that the cell *must be* polarized and non-circular to move (cf. [8]), implying that the distribution of polarization 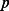 is not axisymmetric. Unfortunately, the developed description does not allow to model the stick-slip motion of cells perpendicular to the adhesive stripes, cf. Fig. 7b, because significant shape deformations are involved in that case.

We will describe the center of mass of the cell by 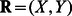 and assume a fixed circular shape, given for the sake of simplicity by a Gaussian approximation 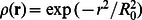, with 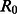 measuring the size of the cell. This choice makes analytical calculations possible. For the case of a homogeneous substrate, for the adhesive sites we assume 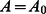 for 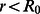, where 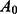 is a solution to Eq. (4). In case of periodic modulations of the substrate’s adhesiveness (e.g. stripes in 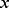-direction as discussed above), these will be approximated by a square wave 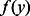 with 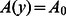 on the adhesive stripes and 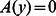 between the stripes. The distribution 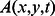 then becomes 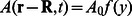 where 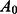 is again a solution to Eq (4). For the following it is enough to use a Fourier expansion and to keep only the two first modes. Here we used the expansion 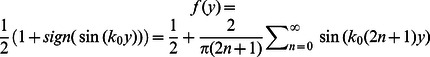.

Assuming 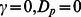, for a stationary cell (i.e. 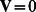) the polarization 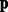 is given to leading order by 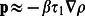, cf. Eq. (2). Similarly, for 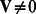, Eq. (2) yields the condition 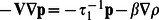 and for small 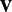 we obtain
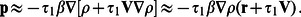
(11)


Hence, to leading order the polarization is just shifted by the amount 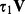. Correspondingly, the net polarization 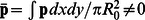 for 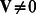.

### Estimate for the Velocity

Here we show how the velocity of a stationary moving cell can be estimated by the use of the circular approximation discussed above. Assuming a solution of the form 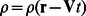 and multiplying Eq. (1) by 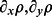, after integration over the entire domain we obtain for the components of the center of mass velocity: 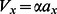, 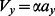, where for a round cell
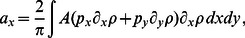
(12)

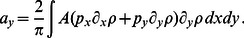
(13)


Let us consider a cell moving along the 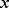-direction, and no substrate modulation. Then 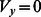 and 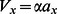, where 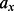 can be explicitly integrated (using the expression (11) for 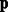) to yield
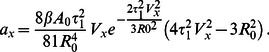
(14)


Substitution into 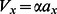 immediately shows that one of the roots is 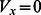, corresponding to the stationary cell. Additional possible roots are given by the equation
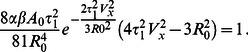
(15)


This equation has additional solutions only for a finite velocity above a certain critical value. To see that, we introduce the dimensionless velocity 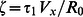 and the dimensionless driving parameter 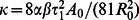. Then Eq. (15) assumes a simple dimensionless form
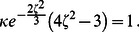
(16)


**Figure 9:**
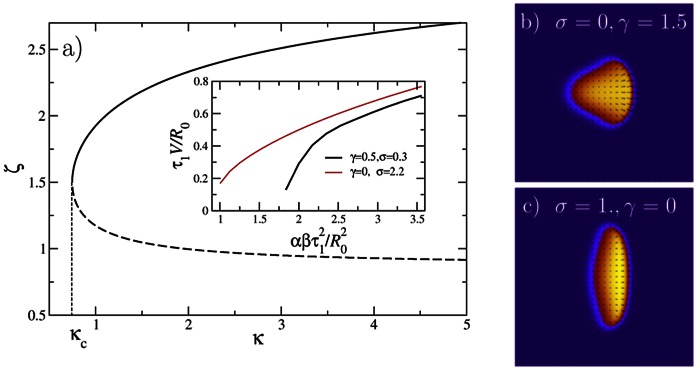
Subcritical onset of motion and stationary cell shapes. Panel a) displays the normalized velocity 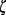 vs. the normalized driving force 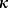. The main plot shows the solutions of Eq. (16), the solid line corresponding to the stable moving branch and the dashed line to the unstable branch. The inset shows results obtained by numerical solution of the full model for 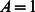, 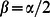, 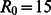, 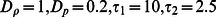 and for different values of 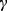 and 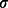 as indicated. b) A typical stable moving shape corresponding to 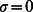 and 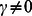. c) A typical stable moving shape corresponding to 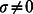 and 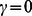.

For 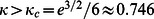, Eq. (16) has two roots, with a minimal finite velocity of 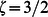. Fig. 9 shows these two branches, the stable upper branch as the solid curve and the lower unstable branch as the dashed curve. This result is in qualitative agreement with the numerical solution of the full model, concerning bistability and the finite velocity gap. However, the numerical values for the dimensionless critical driving parameter 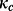 are off by approximately a factor of two due to our approximations, especially due to the assumption of fixed round shape. For comparison, the Inset to Fig. 9 presents the cell velocity vs. the propulsion (both dimensionless again) obtained by direct numerical solution of Eqs. (1)–(6). The black curve is for keratocyte-like parameters (with 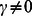 mimicking actomyosin bundle formation at the cell’s rear). The red curve is without this term, but with a higher value of 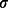, describing the overall contraction by myosin - cf. the discussion above after Eq. (2).

### Why does the Cell Move along the Stripes?

Using the circular approximation we can rationalize why cells move along the stripes in case of a steady-state motion, cf. Fig. 7a. We account now for the fact that 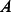 depends on 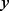. As mentioned above, we will keep only the first two terms in the square wave expansion of the substrate pattern. Expanding the result for 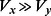, i.e. motion almost along the stripes in 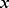-direction, and inserting the result for 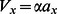 into 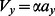, one obtains a single equation for 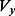:
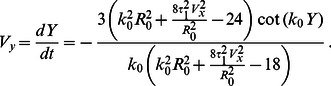
(17)


For 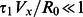, this reduces to
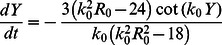
(18)


Note that Eq. (18) describes an overdamped motion of a particle in a periodic potential - given by the stripe pattern characterized by the wavenumber 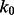. The cell migrates to the center of the stripe if 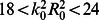 and in between the stripes if 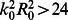. This is consistent with the numerical solution of the full model, cf. Figs. 7a and 8: the cell’s centroid migrates to the center of the stripe for small 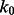 (large period of the stripes) and moves between the stripes for larger 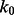.

### Effect of Adhesion Strength and Motor Activity on Cell Velocity and Shape

To further examine our model, we investigated the speed of the cell and its aspect ratio in more detail. Experimentally, these quantities were studied for the steady motion of keratocytes in Ref. [11] [cf. Fig. 8C,E therein], as a function of two parameters: the adhesiveness of the substrate (changed experimentally by varying the grafting density of RGD-peptide-containing block-copolymers, RGD inducing specific cell adhesion via binding to integrin) and the amount of active motors (changed experimentally by using blebbistatin, a myosin inhibitor, and calyculin A, a myosin promoter).

**Figure 10:**
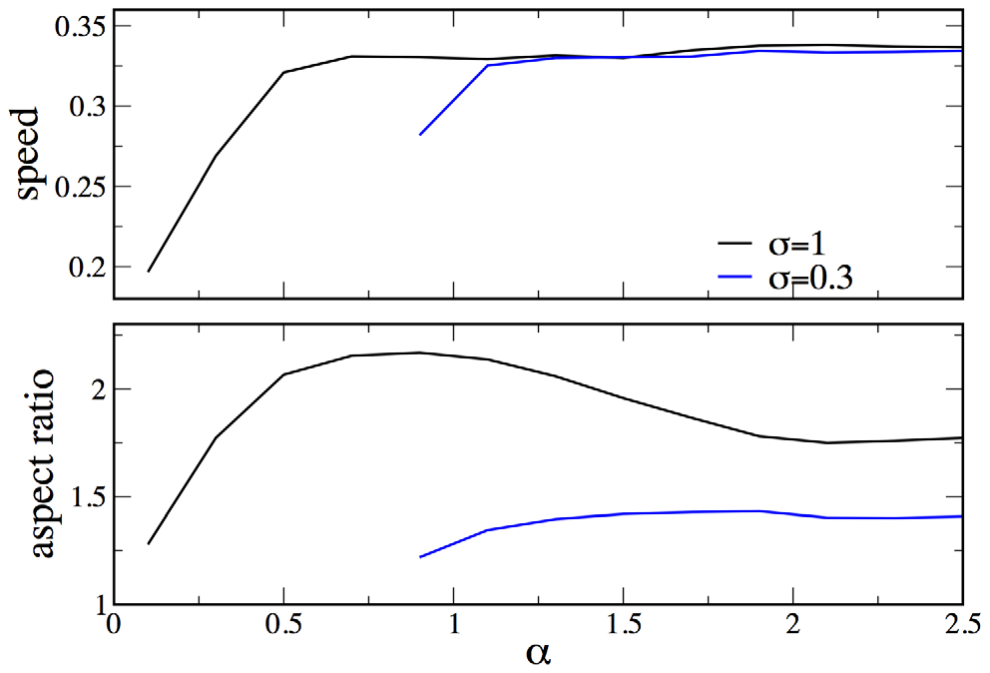
Effect of adhesion strength and motor activity on the velocity and shape. The upper panel shows the cell’s velocity as a function of the propulsion parameter 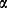 for fixed 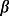, mimicking an increase of the substrate adhesiveness as explained in the text. Two different levels of the contractile motor activity have been investigated, modeled by different values of the parameter 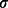. The lower panel displays the corresponding aspect ratios (see Ref. [8] for its definition). Parameters as in Fig. 2, except 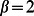, 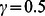.

We performed similar studies within our modeling framework, cf. Fig. 10. In our model, we can assume that the adhesive contact density 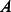 is normalized to the area per grafted ligand. Thus, within this interpretation, the propulsion parameter 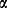 will increase as a function of the coverage/grafting density, while everything else remains unchanged. Note that one can still separate the effects of adhesion and actin polymerization, as the parameter 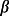 contains only the polymerization rate: increasing 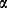 with 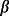 fixed corresponds to increasing the number of grafted ligands, while simultaneous increase of 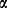 and 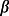 is associated with faster actin turnover.

The upper panel of Fig. 10 shows the cell’s velocity vs. the propulsion parameter 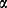 for fixed 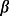, hence mimicking increasing adhesiveness, for two different values of the motor contraction strength. The lower panel displays the respective aspect ratio (i.e. the ratio of the effective cell width to length, see Ref. [8] for our definition). These results can be compared to the experimental data in Ref. [11], Fig. 8C,E: as in the experiments, decreasing the motor activity (i.e. decreasing the contraction parameter 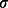) leads to a shift of the adhesion-mediated velocity increase towards higher values of adhesiveness. Also as in the experiments, the aspect ratio shows a maximum as a function of the adhesiveness. For the case of myosin inhibition (lower value of 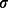), the aspect ration is practically independent on the adhesiveness.

## Supporting Information

Movie S1 **steady motion.**
(AVI)

Movie S2 **stick-slip motion, cf.** **Fig. 2** **.**
(AVI)

Movie S3 **cell bounces off the step in adhesion, cf.** **Figure 6A** **.**
(AVI)

Movie S4 **motion on the substrate with alternating stripes of high/low adhesiveness: motion parallel to the stripes, cf.** **Fig. 7a** **.**
(AVI)

Movie S5 **motion on the substrate with alternating stripes of high/low adhesiveness: motion perpendicular to the stripes, cf.** **Fig. 7b** **.** In all the movies, RGB (red/green/blue) color channels encode the phase field (red), the adhesive contacts (green) and the absolute value of the orientation field (blue), respectively. Hence high adhesive contacts are yellow, highly oriented regions are purple. In the case of substrate modulations, blue color encodes high adhesiveness regions, black color encodes low adhesiveness regions.
(AVI)
